# Editorial: Parents with mental and/or substance use disorders and their children, volume II

**DOI:** 10.3389/fpsyt.2022.1020660

**Published:** 2022-09-13

**Authors:** Joanne Nicholson, Jean Lillian Paul, Joanne Riebschleger, Anja Wittkowski

**Affiliations:** ^1^The Heller School for Social Policy and Management, Institute for Behavioral Health, Brandeis University, Waltham, MA, United States; ^2^Division of Psychiatry I, Department of Psychiatry, Psychotherapy and Psychosomatics, Medical University Innsbruck, Innsbruck, Austria; ^3^School of Social Work, Michigan State University, East Lansing, MI, United States; ^4^Division of Psychology and Mental Health, Faculty of Biology, Medicine and Health and the Manchester Academic Health Science Centre, School of Health Sciences, The University of Manchester, Manchester, United Kingdom

**Keywords:** parents, mental health, substance use disorders, children, families, family-focused practice

## Introduction

The first Frontiers eBook compilation of Research Topic articles on Parents with Mental and/or Substance Use Disorders and their Children, published in 2020, included 27 papers, with over 100 contributors from 15 countries. Investigators employed diverse designs and methods to explore the experiences of parents and their families, and to develop and test interventions. While the prior Research Topic was a significant contribution to the field, families living with parental mental and/or substance use disorders remain vulnerable. The present, second volume of papers on this Research Topic fills gaps identified in the first volume, and moves the field forward by highlighting significant relationships and experiences of key stakeholders; the description and application of conceptual models and frameworks; recent innovation in intervention development, adaptation, testing, and sustainability; shifts in policy and practice paradigms toward more integrated models; and further developments in the research process, measures, and methods, particularly given the impact of the COVID-19 pandemic on parents, families, and practice.

The 34 articles in volume II of this Research Topic represent the work of 151 authors from 13 countries, with reviewers from many more, contributing to cutting-edge knowledge and identifying next steps in research, policy, and practice. Rich material is provided as supplements to several of the papers, which readers are encouraged to explore. The articles reflect progress in the field, in the development and application of theory, and intervention specification, sustainability, and impact. Contributions have shifted from describing prevalence to exploring solutions to supporting families, parents, children, and professionals at both policy and practice levels. Several groups of investigators who contributed protocol papers to volume I have summarized their findings in volume II. The research measures and methods papers in volume II provide evidence of greater stakeholder involvement in research, as co-designers and collaborators. There is increasing focus on improving outcomes for adults with mental and/or substance use disorders who are parents, as well as for the children of parents with mental illness (COPMI) across the lifespan, from considering parenthood, to pregnancy and the perinatal period, to adulthood. Colleagues are reporting on the longer-term impact of policy and practice paradigm shifts promoting the identification of the needs of whole families and collaborative efforts to address them. Innovative solutions to the challenges facing whole families may require the support of the larger context and communities in which families reside—“the village.” Families may benefit from the support of both professional and natural resources in their “village,” accessed formally through service delivery channels and informally through family, neighborhood, and community networks.

## Significant relationships and impact

Important, comprehensive review papers contribute to the state of our knowledge. Radley et al. in the UK provide a scoping review of interventions relevant to parents with psychosis, focusing on five groups of diverse intervention components, from talking about to improving parenting skills and experiences, to support for the whole family. The authors underscore the need for RCTs, and the need to identify components effective in changing outcomes for both parents and children. In their systematic review, Reid et al., also from the UK, provide evidence for the relationships between experiences of abuse and maternal suicide ideation, attempted suicide and death, focusing on outcomes for mothers and the perinatal period. They recommend that women with experiences of domestic violence or childhood abuse be identified and provided emotional and practice support during this crucial period. Children's experiences of stigma-by-association are the focus of a systematic mixed studies review by Dobener, Fahrer, et al., investigators in Germany. The authors provide a comprehensive framework of identified aspects of stigma related to parental mental illness and group these into four dimensions (i.e., experienced, anticipated, and internalized stigma and structural discrimination); the importance of anti-stigma interventions and campaigns is emphasized. The potential for expressed emotion to contribute to the transgenerational transmission of mental disorders is examined by Fahrer et al. also in Germany. Their systematic review highlights the dearth of studies on expressed emotion in families in which a parent has a mental illness.

## Experiences of key stakeholders

Fathers are the focus of a single paper in this volume, in which Doi et al. in Japan examine the relationship between workplace and community social capital, and fathers' postpartum depression and anxiety. Community social capital (i.e., social support and resources) was found to be inversely related to symptoms of depression and anxiety, suggesting the potential benefit of promoting paternal social support in the perinatal period. Sabella et al. in the USA employ life story qualitative research methods to explore the experiences of young adult parents with serious mental health conditions. Young adult parents were actively involved as researchers in this community-based participatory research study in which participants described their challenging but motivated parenting journeys. Young adults are also the focus of the study by Villatte et al. in Canada regarding the perceived social support of youth whose parents have a mental illness. Participants described themselves as important sources of support for their parents, and emphasized the need for having other support figures in their lives, a potential target for intervention. Gregg et al. compare expressed emotion and attributions in parents with and without serious mental illness. Parents with schizophrenia exhibited significantly more hostility and criticism toward their children, and less warmth, and made more child-blaming attributions. These findings suggest targets for intervention with parents and families.

## Conceptual models, frameworks, and program theory

Reupert et al. place the notion of “the village” in the context of Bronfenbrenner's ecological theory to underscore the importance of promoting the capacity at all levels (e.g., individual, family, services, government) to provide support and guidance to families living with adversity. They call for further research to explore ways in which village concepts and components may play out in diverse settings with diverse families to develop interventions and evaluate impact. In-depth interviews by Bauer et al. with program implementers inform theory development, illustrating the interconnectedness between changes that need to co-occur in practitioners, parents, and children, and fragmented health systems to enable practitioners to focus on parents' strengths. Drawing from a realist approach and complex systems thinking, the authors link contextual factors with action mechanisms to disrupt the status quo and transform practice. Family-focused practices support adults in their parenting role and mental health recovery, and focus on protecting children and promoting their resilience (Allchin et al.). These investigators engaged stakeholders within adult mental health services to inform the development of a model of key elements influencing the sustainability of a particular intervention, Let's Talk about Children, and, ultimately, a sustainability model for family-focused practice, placing the work in a wider context. The authors underscore the potential benefit of recognizing the parenting status of adult clients to benefit parents, children, and families.

## Intervention development, adaptation, testing, and sustainability

Articles in this volume regarding interventions focus on the engagement of and outcomes for children, parents and families. Hagström analyzed narrative structured interviews with children and parents regarding their experiences in a grief support camp in Sweden for families affected by a parent's suicide. Parents and children reported the benefits of a psychoeducational approach, open communication, and opportunities to connect with others with similar experiences, which contributed to destigmatization of their experiences. Vetri et al. conducted a formative evaluation, examining children's, parents', and workers' perceptions of bibliotherapy using a book with strategies and activities specifically targeted to the elementary school age group. The authors conclude that bibliotherapy may help children learn concrete strategies for coping with challenges, and help families initiate sensitive discussions when a parent has mental illness. A Norwegian team of investigators investigated the rate and characteristics of children's participation in Child Talks, conducting quantitative and qualitative analysis of electronic patient journal entries by healthcare professionals (Kristensen et al.). While sessions with children were relatively rare, participating children knew more about their parents' illnesses and treatment, suggesting the benefit of studying factors influencing their participation. Petzold et al. report findings from an observational study evaluating adherence to an integrated care program (i.e., “Mommy think of me”) for methamphetamine-related mental disorders (e.g., ADHD, depression) in pregnant women and parents. The 15-session intervention draws from motivational interviewing, psychoeducation, and cognitive behavior therapy. Depression and ADHD were significantly related to lower participation in treatment, underscoring the importance of disseminating integrated care concepts to counter the increasing methamphetamine crisis.

Two groups of investigators report on adaptations of Triple P Positive Parenting Program resources. Outcomes of implementing the Triple-P Self-Help Workbook with guidance and support in 10 sessions with parents with psychosis were investigated by a team in the UK (Wolfenden et al.). Improvements in mental health, parenting and child behavior measures were reported and maintained by parents completing all 10 sessions. The authors provide preliminary evidence that symptoms of psychosis may be reduced by improving family functioning. A second team of UK researchers studied the feasibility and acceptability of delivering the Baby Triple-P Positive Parenting Program (BTP) to mothers with severe mental illness in an inpatient Mother and Baby Unit (Wittkowski et al.). They compared characteristics, participation and outcomes for women in two conditions: (1) treatment as usual and (2) BTP in addition to treatment as usual. The authors provide a thorough overview of their study procedures, preliminary findings, and lessons learned to inform wider implementation in existing perinatal mental health services and a future, larger RCT.

Two papers provide insight into the perspectives of families and practitioners implementing the Family Talk intervention in 15 sites in Ireland (Furlong et al.; Mulligan et al.). The two studies reported here are nested within an RCT. The vast majority of families reported substantial benefits from participating in Family Talk (e.g., increased confidence, improved communication), and identified key facilitators (e.g., non-judgmental clinician) and barriers to participation (e.g., stigma). The authors provide a comprehensive discussion of implementation issues, with recommendations for addressing them across phases of participation. Mental health clinicians and managers were interviewed to investigate their experiences implementing Family Talk and perspectives on longer-term sustainability. Participants described key factors to successful implementation, including organizational support, clinician skills, and appreciating the benefits for families. The benefits of a structured, manualized approach are highlighted, along with a call for the development of a multi-level public-health response to address societal and systemic barriers to change.

The adaptation of Let's Talk about Children (LTC) in the Massachusetts USA adult mental health services context—the ParentingWell Practice Profile—is described in detail by Nicholson et al., who delineate program theory and action mechanisms. Supplementary materials provided with this article include the ParentingWell Practice Profile, a Workbook of activities for practitioners and parents, and self-assessment resources for use in training, supervision, and coaching. The development and adaptation of LTC in various contexts, alongside the developing evidence base, is documented by Allchin and Solantaus. Drawing from their review of the literature regarding LTC, the authors identify three forms of LTC, with outcomes related to parents, and family and child wellbeing and evidence of effectiveness in implementation contexts. The contribution of this paper lies in the use of LTC as an example of an evidence-based practice developed in the context in which it was implemented, rather than the academic setting or laboratory, to guide and inspire future innovation, and support sustainability over time.

A Research Topic in psychiatry or public health in 2022 would not be complete without an article on the impact of the COVID-19 pandemic on policy, research and practice. Obradovic and Nicholson provide a perspective on pandemic-related adaptations in family-focused service delivery given the dramatic changes in people's lives, with implications for research measures, methods, and outcomes. The authors couch their perspective in the EASE Framework to highlight consequences for engagement, assessment, support, and education of family members. Treatment targets and timeframes may have shifted, and routine outcomes may have to be re-evaluated. Hopefully, pandemic-induced changes in access to and participation in services and research (e.g., virtual strategies) will help to promote engagement, and address inequities and disparities.

## Cross-sector and systems level approaches

Concerns have been raised about the potential over-representation of parents with mental health and substance use disorders in the child welfare system. Effective supports for families living with parental challenges may well require cross-sector efforts as well as within-sector or within-system identification and response. Vis, Lauritzen C, Havnen, et al. in Norway tested their hypotheses regarding child protection and welfare reports in a case file study. Reported concerns about mental illness and substance abuse problems were substantiated in over half of the cases. Services were provided in just over a third of the cases, and were not more or less likely in cases about mental illness and substance abuse than in other types of cases. A second study by these researchers focuses on the involvement of children in child welfare and protective services investigations (Vis, Lauritzen, Christiansen et al.). In situations in which the parent's mental health was a concern, conversations with children were conducted much less frequently than in situations when the child's problem was the focus of the report. Investigations based on concerns regarding parental mental health took more time and effort than other investigations. The authors call for a national knowledge-based system and a focus on children's needs in child welfare. In a third study by this team, the investigators explored the extent to which children were identified in the records of patients with mental illness and substance use disorders (Reedtz et al.). The identification of minor children has increased since the Norwegian Health Personnel Act (2010), with over half identified in 2020. In slightly fewer than one-third of the cases, health personnel provided support to children. The authors conclude that children remain unidentified and underserved, and recommend enhancements in the skills of clinicians.

Everts et al. evaluate the implementation of the mandatory identification of the children of adult patients receiving mental health services in the Netherlands. The Dutch COPMI check is part of the first step in a five-step protocol, in which parental mental health is a warning sign of risk for child abuse. Patient files were examined to extract data for the study, which were complemented by focus group discussions with professionals. For the majority of adult patients, the COPMI check tool was not used. The authors recommend that a shift to a “needs/support” focus could be geared to helping children when there is no immediate threat to their safety.

An integrated family approach in mental health services often requires the collaboration of professionals from adult and child mental health services to support family members and prevent the intergenerational transmission of psychopathology (Stolper et al.). This reflects a paradigm shift from an individual practice model to a family centered model, for which many professionals are unprepared. Group interviews with professionals were conducted to explore their experiences working with families and identify the challenges in implementing a family centered model. Differences in perspectives (i.e., adult service provider vs. child provider) and loyalties contributed to challenges in setting treatment targets and in information exchange. A focus on the whole family, flexible treatment planning, and multidisciplinary consultation were perceived as contributing to success.

## Contributions to research measures, methods, and processes

Several papers in this volume highlight comprehensive measurement development processes. Riebschleger et al. describe the development and initial testing of the Youth Mental Health Literacy Scale for ages 11–14. Drawn from theoretical perspectives on mental health literacy, with input from diverse stakeholders, further psychometric analyses suggested refinements in subscales and reductions in items. The result is a scale that can be useful with the general population as well as with youth with a family member with mental illness in assessing needs and testing the effectiveness of mental health literacy programs. Dobener, Stracke, et al. hone in on the challenges conveyed by stigma in developing the Children of Parents with Mental Illness—Stigma Questionnaire (COPMI-SQ) for youth aged 12–19 years. Based on extensive literature review, and discussions with experts and youth, the investigators report pilot data on the measure's psychometric properties. They describe next steps in reliability and validity testing. These measurement developments will contribute to rigorous research on the experiences and needs of youth, and to building the evidence based of effective prevention and intervention approaches.

Community/stakeholder engagement in research, co-design and co-production reflect cutting-edge approaches to the implementation of research *per se*, as well as to the development, adaptation, and testing of interventions. The facilitated, transdisciplinary process supported by the Ludwig Boltzmann Gesellschaft (LBG) is described by Kaisler and Grill. The governance structure for funded projects included diverse stakeholders—researchers, individuals with lived experience, and an open innovation expert—along with a competence group of young adult offspring of parents with mental illness. The authors highlight the challenges to researchers, including the complexity of the process and the integration of various perspectives and skillsets. Goodyear et al. reported on steps in the co-development and implementation of the “It takes a Village” collaborative practice model to promote child-focused support networks in Austria for families in which a parent experiences a mental illness. They highlight the importance of regional, context-specific solutions in designing care models. A similar co-design, co-development process is detailed by Nicholson et al., as they adapted the Let's Talk about Children model—the ParentingWell Practice Profile—as described above. A specific method for engaging mothers with mental health and/or substance use conditions in research is provided by Zisman-Ilani et al. The Virtual Community Engagement Studio (V-CES) approach was developed and piloted in the USA during the pandemic, when accessible virtual strategies for actively engaging research participants and patients became essential. The V-CES toolkit is provided as supplementary material, and offers a step-by-step, accessible, supportive approach to mothers and others from underserved or marginalized populations as research collaborators.

Several research teams highlight the importance of using data to support the development, implementation, and sustainability of preventive and supportive interventions for children, youth, and families living with parental mental illness. The team collaborating on the Danish High-Risk and Resilience Study continued in the third wave of assessment to collect a wide range of data on multiple domains of children's functioning over time (Thorup et al.). Their goal is to develop a comprehensive understanding of the developmental trajectories of children at familial risk for mental illness to identify optimal time points and domains for targeted preventive and early intervention approaches. Finally, Takalo et al. provide an example of the use of data from multiple sources in Finland, including population level, regional, and local data, to inform the implementation of the collaborative Let's Talk about Children Service Model in a pilot region. The inclusion of diverse services sectors, guided by a collective impact framework, provides the context for the sustainability of stand-alone interventions.

## Next steps

Articles in this volume represent innovation in approaches and advances in our thinking about how best to work together with parents with mental and/or substance use disorders and their families to ensure positive outcomes for all family members. Challenges remain in specifying interventions and their action mechanisms in greater detail, to facilitate rigorous research with a focus on outcomes for both adults and children. Innovative perspectives on adapting and studying interventions in new contexts and sustaining them over time suggest the importance of further research, not only focused on outcomes *per se*, but on the characteristics of collaborations, contexts, and communities that support the scaling up and out, and sustainability of these efforts. Next steps in the field must focus not just on what to do, but how to do it—how to engage stakeholders effectively—parents, children, practitioners, policymakers, funders, legislators—as partners in this endeavor. The development of initiatives and collaborations within and across countries underscores a growing commitment to promoting positive outcomes for whole families, and offers increasing opportunities for researchers, policymakers, practitioners, and family members to work together to achieve this goal.

We would like to dedicate this volume to Dr. Mary Seeman, MDCM, FRCPC, DSc, Professor Emerita in the Department of Psychiatry at the University of Toronto. Dr. Seeman is a tireless, committed leader in the field, focusing in particular on gender, psychosis, and the experiences of women and mothers with serious mental illnesses. As a co-editor of each of the three editions of Parental Psychiatric Disorder, Dr. Seeman's contributions have spanned decades (1). She has personally supported many of us in our professional development and research careers. Dr. Seeman's many seminal papers bring attention to the needs of women with schizophrenia and treatment considerations, including the importance of addressing reproductive issues and effective parenting. It is her contention that comprehensive treatment of schizophrenia in women means remembering that all women of childbearing age are potential new mothers, and that women with schizophrenia who are parents benefit from ongoing support (2). In 2013, Dr. Seeman wrote that “useful services for parents with schizophrenia need to bridge the adult/child mental health divide and provide family-centered care with full interagency cooperation” (p. 19), citing references from the early 2000's, and presaging conclusions and recommendations in this current volume, nearly a decade later. Dr. Seeman provided thoughtful reviews for many of the papers in the current volume, for which we are grateful. Dr. Seeman's commitment and contributions inspire us to move forward with this important work.

## Author contributions

JN initiated the Research Topic. JN, JP, JR, and AW were topic editors and wrote the manuscript. All authors contributed to manuscript revision, read, and approved the submitted version.

## Conflict of interest

The authors declare that the research was conducted in the absence of any commercial or financial relationships that could be construed as a potential conflict of interest.

## Publisher's note

All claims expressed in this article are solely those of the authors and do not necessarily represent those of their affiliated organizations, or those of the publisher, the editors and the reviewers. Any product that may be evaluated in this article, or claim that may be made by its manufacturer, is not guaranteed or endorsed by the publisher.
